# VITAMIN E INTAKE AND FOOD SOURCES IN ADOLESCENT DIET: A CROSS-SECTIONAL POPULATION-BASED STUDY

**DOI:** 10.1590/1984-0462/2021/39/2019295

**Published:** 2020-12-14

**Authors:** Karyne Sumico de Lima Uyeno Jordão, Daniela de Assumpção, Marilisa Berti de Azevedo Barros, Antonio de Azevedo Barros

**Affiliations:** a Universidade Estadual de Campinas, Campinas, SP, Brazil.

**Keywords:** Adolescent, Vitamin E, Food consumption, Health surveys, Adolescente, Vitamina E, Consumo alimentar, Inquéritos epidemiológicos

## Abstract

**Objective:**

To assess vitamin E intake and its relationship with sociodemographic variables, and to identify the main dietary sources of the nutrient in the diet of adolescents.

**Methods:**

This is a population-based cross-sectional study that used data from 891 adolescents living in Campinas, SP, participating in ISACamp 2014/15 (Health Survey) and ISACamp-Nutri 2015/16 (Food Consumption and Nutritional Status Survey). The nutrient intake averages were estimated using the Generalized Linear Model, adjusted for the total energy of the diet. Dietary sources of vitamin E were identified from the calculation of the relative contribution.

**Results:**

The average vitamin E intake was 3.2 mg for adolescents aged 10 to 13 years and 3.5 mg for those aged 14 to 19 years, results far below the recommended values of 9 and 12 mg, respectively. The prevalence of inadequacy was 92.5%. ­Ten ­foods/­food groups represented 85.7% of vitamin E present in the adolescents’ diet; the vegetable oils group accounted for more than a quarter of the contribution (25.5%), followed by cookies (9.1%) and beans (8.9%).

**Conclusions:**

There were a low intake and a high prevalence of inadequate vitamin E intake among adolescents in Campinas, with vegetable oil as the main source. For the total number of adolescents, almost 33% of the nutrient content was derived from foods of poor nutritional quality such as cookies, packaged snacks, and margarine. The results of this study can guide public health actions that aim to improve the quality of adolescents’ diets.

## RESUMO

**Objetivo:**

Avaliar a ingestão de vitamina E e sua relação com variáveis sociodemográficas, e identificar as principais fontes alimentares do nutriente na dieta de adolescentes.

**Métodos:**

Trata-se de estudo transversal de base populacional que utilizou dados de 891 adolescentes residentes em Campinas, SP, participantes do ISACamp 2014/15 (Inquérito de Saúde) e ISACamp-Nutri 2015/16 (Inquérito de Consumo Alimentar e Estado Nutricional). As médias de ingestão do nutriente foram estimadas por meio de modelo linear generalizado, ajustado pela energia total da dieta. As fontes alimentares de vitamina E foram identificadas pelo cálculo de contribuição relativa.

**Resultados:**

A ingestão média de vitamina E foi de 3,2 mg para os adolescentes de 10 a 13 anos e de 3,5 mg para os de 14 a 19 anos, resultados bem inferiores aos valores recomendados de 9 e 12 mg, respectivamente. A prevalência de inadequação foi de 92,5%. Dez alimentos/grupos alimentares representaram 85,7% da vitamina E presente na dieta dos adolescentes; o grupo dos óleos vegetais totalizou mais de um quarto da contribuição (25,5%), seguido dos biscoitos (9,1%) e dos feijões (8,9%).

**Conclusões:**

Observou-se baixa ingestão e elevada prevalência de inadequação do consumo de vitamina E nos adolescentes de Campinas, apontando o óleo vegetal como principal fonte. Para o total de adolescentes, quase 33% do teor do nutriente derivava de alimentos de má qualidade nutricional como biscoitos, salgadinhos de pacote e margarina. Os resultados deste estudo podem direcionar ações de saúde pública que objetivem melhorar a qualidade da dieta dos adolescentes.

## INTRODUCTION

Cardiovascular diseases represent the primary cause of morbidity and mortality in Western countries.^1^ Atherosclerosis has been shown to start in childhood and progress during life.^2^ ^,^ ^3^ Studies carried out with children and adolescents show that risk factors acquired in these phases tend to remain in adulthood.^3^ Therefore, it is necessary to identify risk factors for coronary heart disease early to establish a primary prevention approach.^4^ ^,^ ^5^

The quality of food during adolescence is very relevant since inadequate eating habits contribute to the emergence of chronic non-communicable diseases (NCDs) such as obesity, type 2 diabetes mellitus, and cardiovascular diseases.^6^ ^,^ ^7^ One of the most important factors to prevent lipid peroxidation and atherosclerosis is the intake of antioxidants.^7^ Thus, the food consumption pattern is one of the determinants of cardiovascular risk, considering that the increased intake of non-hydrogenated vegetable oils, oilseeds, fish, fruits, vegetables, and whole grains is associated with reduced risk.^8^

The demand for micronutrients increases in adolescence due to the growth process.^9^ Certain nutrients and components of the food matrix have stood out due to their ability to reduce oxidation of free radicals and oxidative stress.^6^ Among them, vitamin E stands out, consisting of a group of eight fat-soluble compounds, which develop specific biological activities, with α-tocopherol being the most potent and abundant antioxidant in tissues, plasma, and low-density lipoproteins (LDL-c).^10^Vitamin E is naturally present in foods of plant origin, especially in whole grains, seeds and oilseeds, and vegetable oils, and it is also found in some foods of animal origin, such as liver and egg yolk.^9^ ^,^ ^10^

For the antioxidant action to be effective in the human body, the diet must be healthy and varied in foods with diverse nutrient sources^11^ since synthetic vitamin E added to fortified or supplemented foods does not result in natural benefits^12^ because it does not have the same biological activity due to the structural complexity of the vitamin E molecule.^9^ ^,^ ^13^

In 2000, the United States Institute of Medicine established recommendations for vitamin E intake by age group through the dietary reference intakes (DRI).^10^ To calculate the estimated average requirement (EAR), the intake quotas for adolescents were extrapolated based on the recommendation for adults, considering body differences and the growth process. The vitamin E EAR for adolescents of both genders is 9 mg for the 10 to 13-year-old age group and 12 mg for the 14 to 19-year-old age group.^10^

Highlighting the importance of vitamin E as a dietary antioxidant and the scarcity of data on its intake, studies that investigate the consumption profile of this nutrient become relevant because it is an antioxidant marker of a healthy diet that is little ingested by the population, which brings health benefits and contributes to the prevention of cardiovascular diseases. Also, the identification of the food consumption profile is a necessary task to guide strategies to promote an adequate and healthy diet.^14^ The objectives of this study were to assess vitamin E intake and its relationship with sociodemographic variables and identify the main dietary sources of the nutrient in the diet of adolescents.

## METHOD

This is a cross-sectional population-based study, using data from the Health Survey in Campinas (ISACamp 2014-15) and the Food Consumption and Nutritional Status Survey (ISACamp-Nutri 2015-16). The surveys collected information from adolescents aged 10 to 19 years old, non-institutionalized, and living in the urban area of the city of Campinas (SP).

The ISACamp 2014-15 sample was obtained by probabilistic sampling, by clusters, and in two stages: census tract and household. In the first stage, a systematic drawing of 70 census tracts was carried out with probability proportional to the size (number of households). The census tracts were ordered by the average income of the heads of households and, subsequently, 14 sectors were selected in each of the five health districts of the municipality.

**Figure 1:**
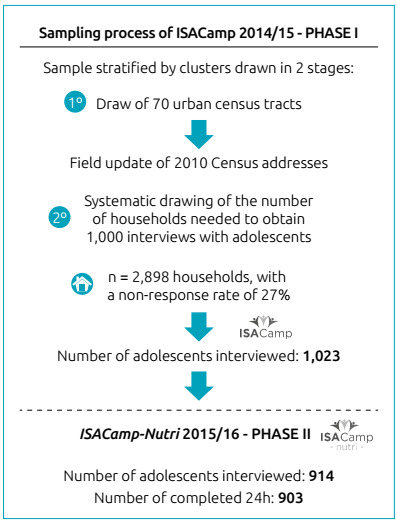
Flowchart of the sample selection process.

The minimum sample size was defined in a thousand adolescents, considering the estimate of a proportion of 50% (p=0.50), which corresponds to the maximum variation for the frequency of the events studied, with a 95% confidence level (z score=1.96), sampling error between four and five percentage points, and a design effect of two.^15^ Predicting a non-response rate of 27%, 2,898 households were randomly selected for interviews with adolescents. In each household, all residents who were between 10 and 19 years old were interviewed (Figure 1). Data collection was carried out by trained interviewers, and the information was entered using a tablet. ISACamp and ISACamp-Nutri were developed together. A team of trained and supervised interviewers carried out a second home visit to invite ISACamp participants to answer a food consumption assessment questionnaire that contained the 24-hour recall (R24h). The filling of the R24h was conducted using the Multiple Pass Method. This structured interview technique aims to stimulate the respondents’ memory and reduce errors that occur in the collection of data on food consumption.^16^ The R24h were applied with the support of a photographic manual. Food/preparations were recorded in units and home measures and, later, quantified in grams or milliliters with the aid of tables of home measures,^17^ ^,^ ^18^ food labels, and customer service. The data were inputted in the Nutrition Data System for Research (NDS-R) software, version 2015 (Nutrition Coordinating Center, University of Minnesota) by trained nutritionists.

The NDS-R uses a database, the Nutrition Coordinating Center Food and Nutrient Database, which contains more than 170 nutrients, 18 thousand foods, and 7 thousand product brands. The NDS-R allows culinary preparations that are not in the database to be inserted in a user’s recipe folder.^19^ After entering the data, all the research records were made consistent.

The dependent variables used in the study were:

- Vitamin E intake (mg/day), estimated based on data from an R24h.
- Dietary sources of vitamin E: the foods eaten by the participants were coded and then organized into food groups or presented separately, considering the main sources of vitamin E. The ten groups/foods that contributed most to the total nutrient in the adolescent’s diet.

To calculate the relative contribution (RC) of dietary sources of vitamin E, the method proposed by Block et al.^20^ using the Equation 1:

$$CR\;=\frac{Total\;vitamin\;E\;of\;the\;food\;(mg)}{Total\;vitamin\;E\;of\;the\;diet\;(mg)}\times 100$$ (1)

Demographic and socio-economic variables were considered as independent variables: gender (male and female), age group in years (10 to 13 and 14 to 19 years old), self-reported race/skin color (white and non-white), education of the head of the household (0 to 4, 5 to 8, 9 to 11, and ≥12 years of study), family income per capita (≤0.5; > 0.5 to ≤1; > 1 to ≤1.5 and >1.5 minimum wage), and whether he/she attends school (no and yes, differentiated by public or private school).

For statistical analysis, the average vitamin E intake was estimated according to the categories of independent variables by age group. Considering the differences in the recommended values of vitamin E, the average intake of the nutrient was calculated for the age groups of 10 to 13 years old and 14 to 19 years old. To find the distribution of vitamin E data, exploratory analysis (descriptive measures, graphs) was performed, and the maximum likelihood estimation method was used to adjust the distributions. Then, graphic techniques and the Akaike Information Criterion (AIC) method were applied to select the distribution with the best fit, showing the range as adequate to model vitamin E intake. The average intake and the respective 95% confidence intervals were estimated using a generalized linear model. To assess vitamin E intake regardless of energy consumption, the model was adjusted for the total energy of the diet (kcal/day), inserted as a continuous variable, according to the waste of Willett et al.^21^

In the analysis, individuals with energy intake below 600 kcal and above 6 thousand kcal/day were excluded. The prevalence of inadequate vitamin E intake was estimated by the cut-off point for EAR, which corresponds to 9 mg for adolescents aged 10 to 13 and 12 mg for those aged 14 to 19 years.^10^ Statistical analyses were carried out using the software Stata version 14.0, in the Survey module, which considers the weights and the sampling design.

ISACamp (Certificate of Presentation for Ethical Appreciation - CAAE No. 37303414.4.0000.5404) and ISACamp-Nutri (CAAE No. 26068214.8.0000.5404) were approved by the Research Ethics Committee of Universidade Estadual de Campinas and by the National Committee of Ethics in Research (CEP/CONEP system). All participants signed the free and informed consent form (FICF), and, for adolescents under 18 years old, the FICF was signed by their parents or guardian.

## RESULTS

Among the 1,023 adolescents interviewed at ISACamp, 109 did not participate in ISACamp-Nutri, 11 refused to complete the R24h, and another 12 were excluded from the present study because they had diets with an energy value below 600 kcal/day (n=10) and above 6 thousand kcal/day (n=2). Thus, data from 891 adolescents aged 10 to 19 years, with an average age of 14.6 years, were analyzed (95%CI 14.4-14.8).

The results of the generalized linear model fitted for the total energy of the diet (waste method) are shown in Table 1. The average intake of vitamin E was 3.2 mg (95%CI 2.8-6) for adolescents aged 10 to 13 years and 3.5 mg (confidence index - 95%CI 3.2-3.8) for those aged 14 to 19 years, results well below the recommended values of 9 and 12 mg, respectively. In the 10 to 13-year-old age group, the average vitamin E intake was significantly higher in girls compared to boys. There was no statistical difference between the average vitamin E intake and the other sociodemographic variables selected for the study.

**Table 1:** Vitamin E intake averages (mg/day) in adolescents, according to age group (in years) and other sociodemographic variables. ISACamp-Nutri, 2015/16.

Variables and categories	10 to 13 years old			14 to 19 years old		
	n	Average^a^[IC95%]	p-value	n	Average^a^[IC95%]	p-value
Gender						
Male^b^	169	2.8 [2.5-3.1]		294	3.4 [3.0-3.7]	
Female	143	3.5 [2.8-4.2]	0.003	285	3.6 [2.8-4.4]	0.392
Total	312	3.2 [2.8-3.6]		579	3.5 [3.2-3.8]	
Race/skin color (self-reported)						
White^b^	169	3.1 [2.7-3.5]		318	3.4 [3.1-3.7]	
Non-white	141	3.4 [3.6-4.2]	0.239	259	3.6 [2.9-4.4]	0.410
Education level of the head of household (in years)						
Up to 4^b^	56	3.6 [2.8-4.4]		121	3.3 [2.7-3.9]	
5 to 8	98	3.2 [1.6-4.8]	0.339	194	3.4 [2.2-4.5]	0.815
9 to 11	94	3.1 [1.5-4.0]	0.252	163	3.5 [2.3-4.7]	0.614
12 or more	54	3.3 [1.7-5.0]	0.593	96	4.1 [2.6-5.7]	0.107
School attendance						
No^b^	11	4.0 [2.6-5.5]		167	3.5 [3.0-4.1]	
Yes, public	247	3.2 [2.9-6.0]	0.203	323	3.5 [2.4-4.6]	0.946
Yes, private	51	3.1 [2.8-6.0]	0.186	87	3.4 [2.2-4.6]	0.774
Family income *per capita* (in minimum wage)						
Up to 0.5^b^	87	3.2 [2.8-3.6]		147	3.3 [2.8-3.7]	
>0.5 to ≤1.0	105	3.4 [2.5-4.3]	0.524	197	3.4 [2.4-4.4]	0.635
>1.0 to ≤1.5	71	3.1 [2.3-4.0]	0.717	119	3.5 [2.4-4.7]	0.395
>1.5	49	3.2 [2.2-4.1]	0.888	116	3.9 [2.5-5.3]	0.209

n: number of individuals in the unweighted sample; 95%CI: 95% confidence interval; ^a^ adjusted by the total energy of the diet; ^b^ reference category used for comparison.

The prevalence of inadequate vitamin E intake was 92.5% (95%CI 90.6-94) in the total population, 91.6% (95%CI 88.1-94.2) in boys, and 93.5% (95%CI 91-95.3) in girls (p=0.358). Adolescents aged 10 to 13 years showed less inadequate intake (p<0.001) compared to those aged 14 to 19 years: 87.7% (95%CI 83.3-91) and 95.1% (95%CI 93-96.7) (data not shown in table).

Table 2 shows that ten food groups represent 85.7% of the total vitamin E present in the adolescents’ diet. For the total population and both genders, the group of vegetable oils provided the highest content of vitamin E ingested. The contribution from ultra-processed foods, such as packaged snacks, stands out, which for the general population ranked fourth and, for girls, reached the second place among the main sources of the nutrient. Important food sources, such as whole grains, oilseeds, fruits, and vegetables, were not listed among the ten groups that contributed most to the total vitamin E.

**Table 2:** Position among the ten main food groups and respective percentage relative contribution to the total vitamin E (mg/day) in the diet of adolescents. ISACamp-Nutri, 2015/16.

Groups/foods	Total population			Male			Female		
	n	Position	RC^a^	n	Position	RC^a^	n	Position	RC^a^
Vegetable oils^b^	3937	1	25.5	2113	1	26.1	1824	1	24.7
Sweet and savory cookies	337	2	9.1	173	3	9.5	164	3	8.6
Legumes	1037	3	8.9	564	2	9.7	473	4	7.9
Packaged snacks	88	4	7.8	35	6	6.2	53	2	9.7
Cereals, breads, cakes, pastas, and root vegetables	3228	5	7.7	1707	4	8.6	1521	5	6.7
Margarine	562	6	6.4	285	5	6.6	277	6	6.1
Industrialized sauces^c^	644	7	5.8	320	7	5.6	324	7	5.8
Processed meats^d^	630	8	3.7	338	8	4.5	292	9	3.6
Milk and milk derivatives^e^	1693	9	3.6	907	10	3.5	786	8	3.7
Bovine meat	582	10	3.4	340	9	4.3	242	10	2.6
Total RC			85.7			83.8			79.4

^a^RC: percentage relative contribution; ^b^includes non-hydrogenated vegetable oils and olive oil; ^c^mayonnaise, ketchup, mustard, tomato sauce, soy sauce; ^d^ Nuggets and luncheon meats such as ham, mortadella, salami, turkey breast, weenie, bacon, and sausage; ^e^includes milk, cheese, and butter.

## DISCUSSION

The main findings of this study were the identification of a high prevalence of vitamin E inadequacy, the verification of the low intake of the nutrient, and the finding that ultra-processed foods, such as cookies, packaged snacks, and margarine, provided almost 33% of the nutrient content ingested by adolescents in Campinas. Also, healthy foods considered critical dietary sources of vitamin E did not contribute in relation to the total nutrient intake.

The dietary profile of Brazilian adolescents has changed over the years. The 2008-2009 Household Budget Survey (POF) shows a decrease in the consumption of traditional and basic foods at the Brazilian table, such as rice and beans, and foods used as culinary ingredients, such as vegetable oils. Still, the data reveal an increase in the consumption of stuffed cookies, soft drinks, and fast foods, which lead to an increase in the intake of free sugars, saturated and trans fat, and a reduction in the consumption of healthy foods.^22^

High prevalence of inadequate vitamin E intake, similar to the result of this study, was observed in POF 2008-2009, totaling more than 99% in boys and girls, with intake averages of 3.8 and 3.9 mg (girls) and 3.7 and 3.5 mg (boys) in the 10 to 13-year-old and 14 to 19-year-old groups, respectively.^22^ In the São Paulo Health Survey (ISA),^23^ vitamin E intake averages of 4.7 mg for women and 4.9 mg for men were identified, both in the 10 to 19 age group and with 99% prevalence of inadequate intake. Studies attribute the high percentage of insufficient intake to the reduced consumption of whole grains, seeds, and oilseeds.^22^ ^,^ ^23^

The vitamin E intake in Brazil was lower than that observed in developed countries, as evidenced in a study carried out in Japan in 2013,^24^ in which the average intake among adolescents aged 10 to 17 years was 8 mg among boys and 7.4 mg among girls. The higher intakes averages found in Japan can be explained by a diet rich in legumes and seeds, such as soy and sesame, as well as fish and vegetable oils.^10^ ^,^ ^25^

In the United States, data obtained by the *National Health and Nutrition Examination Survey* (NHANES 2003/2006) health and nutrition survey showed intake averages below the recommendation, with 6.6 and 7.6 mg for boys aged 9 to 13 and 14 to 18 years, respectively, and 5.6 mg for girls of both age groups.^26^ These averages were higher than those verified in Brazilian studies, as in the results of the present study, POF,^22^ ^,^ and ISA^23^. The prevalence of inadequate vitamin E intake among adolescents in the United States was 83%,^26^ lower than that observed in this study and POF.^22^

The North American food pattern is characterized by a high intake of sugary drinks, fats, fast foods, and ultra-processed foods, responsible for 58% of the total daily caloric intake, to the detriment of the consumption of foods rich in vitamins and minerals.^27^ ^,^ ^28^ Vegetable oils are often used as ingredients in ultra-processed foods and, although they contain vitamin E, the consumption of these foods is associated with the formation of free radicals and an increase in body mass index (BMI).^28^ In Mexico, data from the *Encuesta Nacional de Salud y Nutrición* (ENSANUT, 2012) showed an average intake of 8.7 mg of vitamin E for boys and 7.1 mg for girls aged 12 to 18 years. In ENSANUT, boys (77.4%) had a lower prevalence of inadequacy than girls (93.4%).^29^

The high prevalence of inadequate vitamin E intake raises questions about the overestimation of the recommendations established by EAR, which were based on the prevention of hemolysis induced by hydrogen peroxide since studies show that healthy populations do not reach the recommended values of vitamin E.^30^ ^,^ ^31^

The diversity of dietary patterns that exist between countries explains the differences between the nutrient average intake. Furthermore, the importance of the dietary source of vitamin E is emphasized. The Japanese population has a higher intake of fresh or minimally processed food sources, coming from a diet rich in products based on soy, algae, fish, green tea, mushrooms, vegetables, and fruits, whose consumption is associated with a lower incidence of cardiovascular diseases.^32^ In contrast, the dietary pattern of Americans, rich in ultra-processed foods, is associated with a higher incidence of obesity and other chronic non-communicable diseases.^28^ Thus, in addition to averaging below the recommended intake of vitamin E, an important antioxidant, the consumption of ultra-processed foods can add inflammatory and pro-oxidative components to the adolescent’s diet.

In this study, the main source of vitamin E in the adolescents’ diet was vegetable oils, including olive oil, which represented more than a quarter of the total intake. Sweet and savory cookies appeared in second place in the ranking of the ten main food groups that provided the highest vitamin E content, for both girls and boys. Legumes ranked third for all adolescents, second for boys and fourth for girls. Traditional on the Brazilian table, beans and other legumes have decreased consumption over the years by teenagers.^22^ Besides their essential contribution to the supply of vitamin E in the diet, legumes are also sources of protein, dietary fiber, B vitamins, and minerals such as iron, zinc, and calcium,^11^ essential nutrients for growth and development of the teenager.^9^ The alternation in the consumption of different legumes should be encouraged since it increases the supply of nutrients and, more importantly, brings new flavors and diversity to the diet.^11^

Out of the ten food groups that contributed most to vitamin E intake, five were ultra-processed foods (sweet and savory cookies, packaged snacks, margarine, processed sauces, and processed meats), totaling 32.8% for both genders. The consumption of ultra-processed foods, characterized by high concentrations of energy, saturated and trans fats, sodium and refined carbohydrates,^28^ is associated with systemic inflammation and higher prevalence of overweight and obesity, insulin resistance, cardiovascular diseases, and neoplasms, such as breast cancer in girls.^10^ ^,^ ^33^

Other fresh foods that are sources of vitamins, minerals, and dietary fibers, notably vitamin E, such as whole grains, fish, seeds, and oilseeds,^9^ ^,^ ^10^ appeared as those that contributed less to the total nutrient. To increase vitamin E intake, it is necessary to stimulate the consumption of these foods.

The Dietary Guide for the Brazilian Population (2014)^11^ is a guiding instrument for actions of food and nutrition education of great importance to face chronic non-communicable diseases in the country, which presents a reality marked by extremes of deficiencies and excesses related to food. The Dietary Guide recommends making fresh or minimally processed foods as the basis of the diet, using oils in small quantities to cook food, giving preference to freshly prepared meals when eating out, and avoiding the consumption of ultra-processed foods. Therefore, if the recommendations in the Dietary Guide were followed, vitamin E intake could be adequate.

In Spain, the study Anthropometry, Intake, and Energy Balance in Spain (ANIBES) found that vegetables, fish, and fruits represented 26% of the total vitamin E in the diet of the Spanish population. Vegetable oils and fats had an even more significant contribution to the total vitamin E, reaching 46%. Although this vitamin came from healthier food sources, 72% of Spanish adolescents had a consumption below the recommendations, with an average intake of 7.5 mg of the nutrient.^34^ The vitamin has antioxidant and anti-inflammatory effects, and low consumption is related to a higher prevalence of atherosclerosis, worse lipid profile, infertility, degenerative diseases such as Alzheimer’s and Parkinson’s, inflammatory, pulmonary, cardiovascular diseases, and diabetes.^9^ ^,^ ^12^

Among the strengths of this study, we highlight the analysis of the contribution of food groups to the total vitamin E in the adolescents’ diet, considering the gap in the literature of studies that proposed this investigation. Also, the method to select the studied sample stands out, which is representative of adolescents in the city of Campinas since it is a population-based study. The methodological complexity of the ISACamp survey makes it possible to generalize the sample results to the adolescent population. However, the population and socio-economic reality of Campinas must be considered in comparison with other areas studied. The study applied a single 24-hour recall, which does not reflect the variability of consumption. However, R24h is considered an adequate instrument to assess the average food and nutrient intake when applied on a population basis and different days of the week and months of the year.^35^ Furthermore, the amount of vitamin E present in industrialized products may be under or overestimated since the brands of food products may differ in relation to the nutrient content.

The study found a high prevalence of inadequate vitamin E intake by adolescents, and the intake of this important antioxidant nutrient was much lower than that recommended by DRI. Ultra-processed foods, which add inflammatory and pro-oxidative components to the diet, stood out among the ten food groups that contributed to the total vitamin E intake by adolescents. These findings reinforce the importance of nutritional guidance aimed at adapting the vitamin E intake, but especially the adoption of a diet in which fresh or minimally processed foods and culinary preparations represent the basis of the diet.
